# Device design and applications of the novel JAVELIN peripheral intravascular lithotripsy catheter

**DOI:** 10.1016/j.jvscit.2026.102315

**Published:** 2026-05-15

**Authors:** Andrew Holden, Sameh Sayfo, Michael C. Siah, Mario Trejo, Albert Lee, Joshua J. Popp, Nick E.J. West, John D. Corl

**Affiliations:** a Auckland City Hospital, Auckland, New Zealand; b Baylor Scott & White, The Heart Hospital Plano, Plano, TX; c UT Southwestern, Dallas, TX; d Shockwave Medical Inc., Santa Clara, CA; e The Lindner Center for Research and Education at The Christ Hospital, Cincinnati, OH

**Keywords:** Calcified lesion, Calcium modification, Intravascular lithotripsy, Peripheral artery disease

## Abstract

**Objective:**

Moderate or severe vascular calcification is associated with an increased risk of restenosis and need for reinterventions after endovascular treatment of peripheral artery disease. Further, calcification, whether medial, chronic total occlusions with resistant or calcified caps, or calcific nodules, all increase the potential complexity of the intervention. Current calcium modification devices can be limited due to their ability to cross severely stenosed lesions or are associated with an increased risk of vessel damage and distal embolization. The Shockwave Javelin peripheral intravascular lithotripsy (IVL) catheter has no balloon but uses a single IVL emitter located immediately proximal to the tip of the catheter and is designed to modify and cross calcified high-grade stenoses, subtotal occlusions, and occluded vessels.

**Methods:**

The prospective, single-arm Mini S Feasibility and FORWARD PAD Investigational Device Exemption studies assessed the safety and effectiveness of the novel Javelin peripheral IVL catheter. One hundred ten subjects with angiographic evidence of moderate to severely calcified peripheral artery disease and Rutherford Category 2 to 5 were enrolled from March 2022 to February 2024. Scenarios well suited for the Javelin IVL catheter became evident in this early experience with the device and are described in detail with case examples. Written informed consent for the collection and publication of data was obtained prior to any study-specific requirements.

**Results:**

The primary safety and effectiveness endpoints of the study both met prespecified performance goals: major adverse events (cardiovascular death, clinically driven target lesion revascularization, or unplanned target limb major amputation) at 30 days was 1.1%; technical success (core lab-adjudicated final residual stenosis of ≤50% without flow-limiting dissection) was 99.0%. The study cohort also had significant improvements in residual stenosis and a low rate of serious angiographic complications (1.0%) at final imaging. The Javelin catheter was found to be particularly useful in situations including severely stenosed lesions in smaller diameter infrapopliteal arteries where the fixed diameter due to an absence of a balloon allows for better crossing, calcified nodules, and chronic total occlusions where focal treatment at the tip of the catheter is important.

**Conclusions:**

Although the FORWARD PAD Investigational Device Exemption/Feasibility studies showed a favorable safety and efficacy profile, continued real-world assessment will be necessary to understand the long-term performance of the device and how the Javelin peripheral IVL catheter fits into the broader calcium modification algorithm.

Endovascular treatment has emerged as an option for the treatment of patients with peripheral artery disease (PAD), particularly for those with below-the-knee (BTK) lesions.^1^^,^^2^ However, moderate or severe vessel calcification, present in more than 50% of BTK lesions,^3^ is associated with an increased risk of restenosis and need for reinterventions after endovascular treatment.^4^, ^5^, ^6^ In selecting the appropriate calcium modification strategy for patients with BTK disease, physicians must often consider factors beyond just the length and arc of calcification, typically used to define calcium severity.^7^^,^^8^ Such characteristics include intimal vs medial calcification—the former commonly found in larger arteries and contributing to luminal narrowing, whereas the latter results in increased vessel stiffness and reduced compliance.^3^^,^^9^ The calcific phenotype of BTK disease segments may include chronic total occlusions (CTOs) with resistant or calcified proximal caps^10^ and calcific nodules^11^^,^^12^—either alone or in combination, which further compounds disease and treatment strategy complexity.

Current technologies for modification of peripheral arterial calcification can be limited in certain scenarios: conventional percutaneous transluminal angioplasty (PTA) balloons may struggle to cross severely stenosed calcific lesions^13^ and are only generally effective in mild to moderate calcified lesions; even when effective, the high pressures required to achieve lesion modification may lead to unacceptable rates of vessel dissection and perforation.^14^^,^^15^ Use of dedicated atherectomy tools may also result in vascular damage, leading to dissection and/or perforation,^16^, ^17^, ^18^ and the risk of distal embolization of atherothrombotic debris generally necessitates the use of embolic protection devices.^19^^,^^20^ In contrast, Shockwave intravascular lithotripsy (IVL) uses ultrasonic acoustic pressure waves to modify vascular calcification with an observed low rate of acute vessel injury and embolic complications.^21^, ^22^, ^23^ IVL has demonstrated effective stenosis reduction in calcific lesions in the superior femoral, popliteal, and infrapopliteal arteries,^24^^,^^25^ although balloon-based platforms may also encounter difficulties in crossing nodular or completely occluded lesions.^25^, ^26^, ^27^

### Shockwave Javelin peripheral intravascular lithotripsy catheter

The Shockwave Javelin peripheral IVL catheter (Shockwave Medical Inc) is a novel IVL platform designed to treat “balloon-uncrossable” calcified lesions in the peripheral vasculature in which a guide wire can be passed through but other devices, such as balloons, imaging catheters, etc, cannot cross (Fig 1). The Javelin IVL emitter is similar to the predicate S^4^ IVL catheter’s emitters and has a maximum of 120 acoustic pulses delivered over 12 cycles with a 1-Hz pulsing frequency. However, unlike the balloon-based S^4^ IVL catheter, the single emitter in the Javelin IVL catheter is located at the distal end of the catheter, just proximal to its tip, to allow for localized delivery of acoustic energy shock waves at the tip of the catheter (Fig 2). Bench tests to characterize the acoustic profile of the Javelin IVL catheter have shown that the IVL treatment field effect extends spherically from the emitter itself, extending beyond the catheter tip as well as perpendicularly to the sides where the catheter is in contact with the vessel wall. This differs from a traditional balloon-based IVL platform where the acoustic pressure waves do not extend past the tip because the IVL emitter is positioned within the working length of the balloon. With both Javelin and balloon-based IVL, the pressure output from the emitter decays as a function of distance.

**Fig 1:**
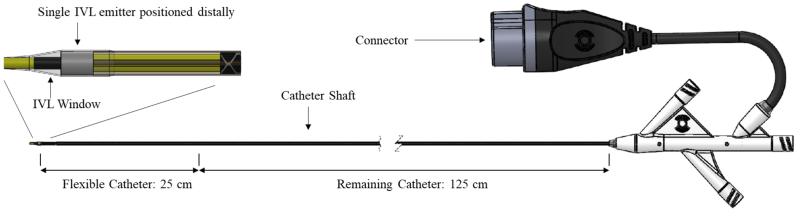
Shockwave Javelin peripheral intravascular lithotripsy (*IVL*) catheter.

**Fig 2:**
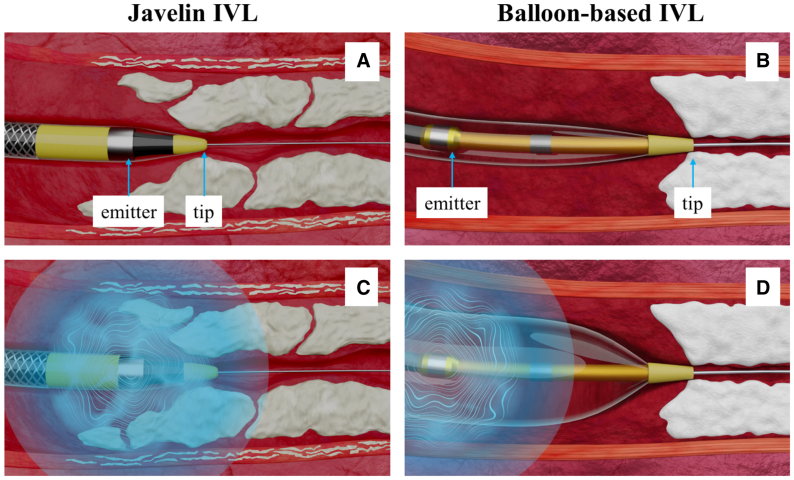
**(A)** The intravascular lithotripsy (IVL) emitter in the Javelin device is located near the tip of the catheter, whereas in balloon-based IVL catheters **(B)** the IVL emitters are within the balloon working length and further away from the tip. **(C)** The *shaded blue circle* denotes the optimal circumferential acoustic energy. This energy propagates beyond the tip of the catheter and decays as a function of distance from the emitter. **(D)** In the balloon-based C2 IVL catheter, although the circumferential energy profile is similar, the acoustic energy does not reach the tip of the catheter because of the emitter position within the working length of the balloon.

The Javelin peripheral IVL catheter is compatible with a 5F introducer sheath and has a constant crossing profile of ≤1.5 mm. Its shaft contains three lumens, one for pressurizing the chamber surrounding the emitter, one to allow flushing between cycles (‘in’ and ‘out’), and a third over-the-wire 0.014” (0.36 mm) guidewire lumen, as well as the lithotripsy emitter and its connections. The catheter’s working length is 150 cm, of which the distal one-third has a hydrophilic coating, and the most distal 25 cm has extra flexibility (Fig 1). We present insights on device usage from the Investigational Device Exemption (IDE)/Feasibility clinical trial as well as case examples of appropriate uses of the Javelin peripheral IVL catheter, based on early clinical experience.

## Methods

### FORWARD investigational device exemption/feasibility studies

The FORWARD IDE/Feasibility prospective, multicenter, single-arm studies assessed the use of the Javelin Peripheral IVL catheter (Shockwave Medical Inc) in the treatment of heavily calcified, stenotic peripheral arteries.^28^ Briefly, the primary analysis cohort included 110 subjects enrolled from March 2022 to February 2024, with the results of the first 90 enrolled being used for the United States regulatory submission.^29^ Subjects were required to have angiographic evidence of moderate to severely calcified PAD and Rutherford category 2 to 5 of the target limb(s) (Supplementary Methods, online only). Study procedural flow included a mandatory postdilatation step, with additional IVL treatment delivery allowed after post-Javelin IVL dilatation if there were areas of >50% residual stenosis. In addition to the option to use commercial IVL catheters, adjunctive therapies including, stents (bare metal/drug-eluting) or drug-coated balloons were permitted at the discretion of the investigator. Written informed consent for the collection and publication of patient data was obtained prior to any study-specific requirements. All sites were required to follow local legal and regulatory requirements for Ethics Committee and Institutional Review Board approvals. The study was conducted in accordance with the Declaration of Helsinki guidelines and Good Clinical Practices.

## Results

Core lab–reported angiographic lesion measurements from the FORWARD IDE/Feasibility studies are in the Table, with most lesions being severely calcified and stenosed, 24.8% (25/101) were eccentric, and 38.0% (38/100) were CTOs. In the IDE/Feasibility studies, 93.0% of Javelin IVL catheters (107/115) were successfully delivered to and crossed the target lesion. The primary safety and effectiveness endpoints of the study at 30 days both met prespecified performance goals^28^: major adverse events (defined as cardiovascular death, clinically driven target lesion revascularization, or unplanned target limb major amputation), 1.1%; and technical success (defined as core lab–adjudicated final residual stenosis of ≤50% without flow-limiting dissection at final angiography), 99.0%. The study cohort also had significant improvements in residual stenosis and a very low rate of serious angiographic complications (1.0%) at final imaging, as described previously.^28^

**Table:** Procedural lesion measurements

Measurements	Javelin only	Javelin + commercial IVL
Core lab–reported angiographic measurements		
BTK lesion^a^	50.6 (39/77)	19.2 (5/26)
Calcification by PARC		
None/mild	2.6 (2/77)	11.5 (3/26)
Moderate	16.9 (13/77)	0.0 (0/26)
Severe	80.5 (62/77)	88.5 (23/26)
Total length of calcification, mm	120.9 ± 88.5 (69)	147.6 ± 86.3 (23)
Lesion length, mm	75.1 ± 62.5 (76)	82.0 ± 50.2 (26)
Long lesion (≥150 mm)	11.8 (9/76)	11.5 (3/26)
Eccentric	24.0 (18/75)	26.9 (7/26)
MLD	0.7 ± 0.7 (74)	0.7 ± 0.7 (26)
RVD	4.0 ± 1.4 (74)	4.8 ± 1.2 (26)
Diameter stenosis	81.9 ± 17.5 (74)	85.6 ± 14.3 (26)
CTO	37.8 (28/74)	38.5 (10/26)
Site-reported procedural characteristics		
Predilatation	3.8 (3/78)	0.0 (0/27)
No. JAVELIN catheters per lesion		
1	92.3 (72/78)	88.9 (24/27)
2	6.4 (5/78)	11.1 (3/27)
3	1.3 (1/78)	0.0 (0/27)
Total no. pulses per target lesion in JAVELIN IVL	82.6 ± 45.4 (77)	108.1 ± 42.1 (27)
Post JAVELIN IVL residual stenosis, %	61.9 ± 24.7 (77)	71.7 ± 16.3 (27)
Postdilatation performed	98.7 (76/77)	96.3 (26/27)
Postdilatation residual stenosis, %	22.8 ± 18.7 (76)	49.0 ± 19.2 (26)
No. commercial IVL devices used per target lesion		
0	100.0 (78/78)	0.0 (0/27)
1	0.0 (0/78)	88.9 (24/27)
2	0.0 (0/78)	7.4 (2/27)
3	0.0 (0/78)	3.7 (1/27)
Total no. pulses per target lesion in commercial IVL devices	NA	230.7 ± 145.6 (27)
DCB used	28.2 (22/78)	74.1 (20/27)
Total no. lesions with stents implanted	25.6 (20/78)	14.8 (4/27)
Final residual stenosis, %	13.7 ± 13.5 (78)	16.1 ± 14.8 (27)
Final total treated length, mm	110.0 ± 58.8 (78)	143.0 ± 76.2 (27)

*BTK*, below-the-knee; *CTO*, chronic total occlusion; *DCB*, drug-coated balloon; *IVL*, intravascular lithotripsy; *MLD*, minimum lumen diameter; *NA*, not applicable; *RVD*, reference vessel diameter; *PARC*, Peripheral Academic Research Consortium.

Data are presented as percent (number/total) or mean ± standard deviation (number).

a BTK lesion is derived from the proximal target lesion locations, which include tibio-peroneal trunk, anterior tibial artery, peroneal artery, posterior tibial artery, and popliteal artery (BTK).

Notably, 25.7% of lesions (27/105) required additional treatment with commercially available balloon-based IVL after Javelin IVL treatment, as shown in the procedural results (Table). The use of balloon-based IVL was more common in lesions that were above the knee (80.8% vs 49.3%), were longer (82.0 vs 75.1 mm), and had greater lengths of calcification (147.6 vs 120.9 mm). After Javelin IVL and postdilatation, operators reported a mean residual stenosis of 49.0% in the lesions that they treated with subsequent commercial IVL, whereas the residual stenosis was 22.8% for the lesions that Javelin IVL and postdilatation alone were considered sufficient. Importantly, the final residual stenosis in both patients receiving Javelin IVL alone and Javelin with commercial IVL were similar (13.7% vs 16.1%). Although the FORWARD IDE/feasibility data represents an early experience within a clinical trial setting, the use of Javelin IVL in real-world scenarios remains to be studied. The following cases provide insights into the potential appropriate applications for the Javelin IVL peripheral catheter.

### Case examples

#### Case 1. A 36-year-old male presented with chronic limb-threatening ischemia with tissue loss (Rutherford class 5, affecting right leg with nonhealing ulcers of great toe and fifth toe)

**Fig 3:**
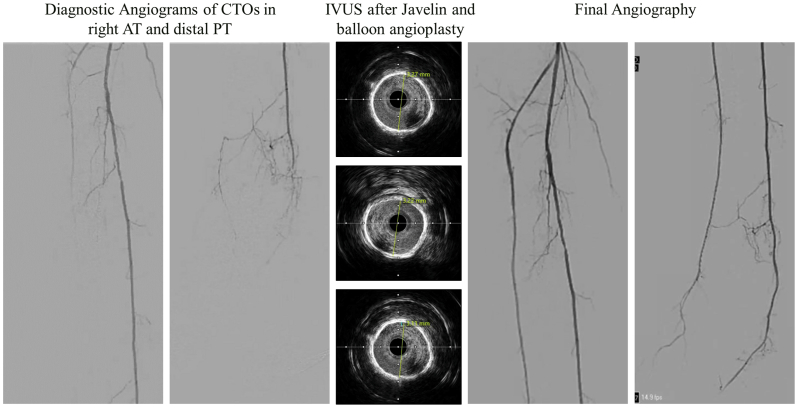
Javelin intravascular lithotripsy (IVL) used to cross and treat calcified chronic total occlusions (*CTOs*) in the right anterior tibial (*AT*) and distal posterior tibial (*PT*) arteries. *IVUS*, Intravascular ultrasound.

Diagnostic angiography revealed CTOs of the midright anterior tibial (AT) and distal posterior tibial (PT) arteries (Fig 3). The PT CTO was crossed with an antegrade approach and treated with 2.0 × 60 mm and 2.5 × 80 mm PTA balloons alone, followed by antegrade crossing of the AT CTO using Javelin IVL. All 120 pulses of the Javelin IVL catheter were used in treating the entire AT artery, extending into the distal dorsalis pedis artery. Post IVL intravascular ultrasound (IVUS) imaging showed no dissection, and further PTA was performed along the entire Javelin-treated segment using 2.0- to 3.0-mm balloons, including long (>200 mm) tapered PTA balloons. After the adjunctive PTA, angiography and IVUS imaging showed indications of a localized dissection in the proximal/mid AT, which was successfully treated with a 3 × 38 Esprit drug-eluting resorbable scaffold.

#### Case 2. An 82-year-old male presented with severe PAD and chronic limb-threatening ischemia with tissue loss (Rutherford class 5, affecting right leg with nonhealing ulcers in toes 1-3)

**Fig 4:**
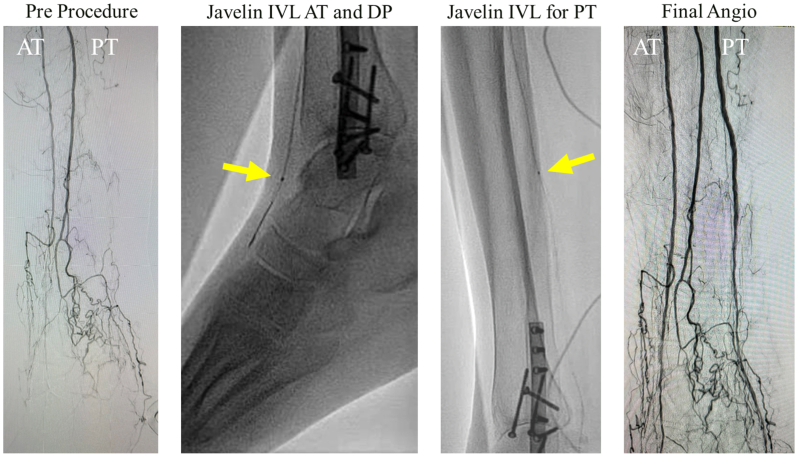
Javelin intravascular lithotripsy (*IVL*) followed by balloon dilatation to treat severely calcified disease in right anterior tibial (*AT*) artery that extended into the dorsalis pedis artery as well as calcification in the right posterior tibial (*PT*) artery. The front of the Javelin catheter can be visualized by the radiopaque IVL emitter (*large yellow arrows*). *DP*, Dorsalis pedis artery.

Diagnostic angiography revealed severely calcified disease in the right AT artery extending into the pedal artery, as well as calcified disease in the right PT artery (Fig 4). After antegrade guidewire crossing, Javelin IVL (120 pulses) was used to modify and cross the right AT CTO and continued into the dorsalis pedis. Following IVL, PTA with 2.0- to 3.5-mm long tapered balloons to consolidate the result. The right PT CTO was crossed in antegrade fashion with a guidewire, and a second Javelin IVL catheter (120 pulses) was used to modify and cross, followed again by PTA with 2.0- to 3.5-mm tapered balloons. Final imaging showed an excellent angiographic result, with restoration of antegrade flow in both AT and PT arteries.

#### Case 3. A 78-year-old male presented with worsening intermittent claudication (Rutherford class 3, affecting right leg) with background of hypertension, chronic kidney disease, and hyperlipidemia

**Fig 5:**
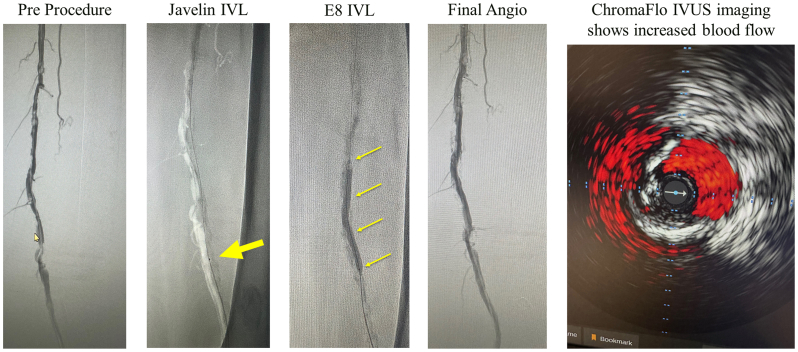
In this case of a multilevel superficial femoral artery (SFA) lesion, the full 120 pulses of the Javelin intravascular lithotripsy (*IVL*) catheter were concentrated in the most calcified regions, and an E8 IVL catheter was used to treat the remaining length of the lesion. *IVUS*, Intravascular ultrasound.

A lower extremity arterial ultrasound showed multilevel stenoses in the right superficial femoral artery with diameter stenoses of 90% and 95% at proximal and distal locations and an overall lesion length of over 200 mm (Fig 5). Preintervention gradient assessed with concomitant pressure measurements between radial and pedal access sheaths was 80 mmHg. Radial access for the Javelin IVL was not possible due to severe tortuosity and multiple loops in the iliac arteries; therefore, AT access was used for a successful retrograde guidewire crossing. Javelin IVL (120 pulses) was used to modify and cross the long lesion, with pulses concentrated in the areas of most severe calcification and severe stenosis. Due to the length of the lesion, a Shockwave E8 IVL catheter (Shockwave Medical Inc) was used after Javelin IVL to treat the remaining length and followed with a 6 × 300 mm drug-coated balloon. Final angiography confirmed residual stenosis of 20%, and restoration of flow was confirmed with ChromaFlo IVUS imaging (Philips Volcano). Postintervention gradient measurements between radial and pedal access sheaths were reduced to 10 mmHg.

## Discussion

The novel Javelin peripheral IVL catheter represents an evolution in the field of complex calcific lesion management: although contemporary balloon-based IVL platforms have been shown to be highly effective in modifying vascular calcification in iliac, femoropopliteal, and infrapopliteal territories,^24^^,^^25^ they have sometimes struggled with ability to cross the most complex calcific lesions without additional vessel prepreparation,^26^^,^^30^^,^^31^ including the use of atherectomy,^16^, ^17^, ^18^ potentially reducing some of the benefits of using a device associated with much lower rates of vascular complication.^21^, ^22^, ^23^ The performance of Javelin IVL in the highly complex patient and lesion population in the FORWARD PAD IDE/Feasibility studies confirms overall safety and efficacy, including low adverse event and dissection rates.^24^^,^^25^^,^^32^ Further, early experience, both in these studies and since commercial rollout, have identified the following potential scenarios of unmet need in which Javelin IVL may be particularly advantageous.

### Balloon-uncrossable lesions and chronic total occlusions

Javelin IVL was originally designed specifically for the treatment of calcific high-grade stenoses and occlusions, particularly for those lesions crossable by wire but not by other devices such as balloons or even imaging catheters.^26^ Although current treatment algorithms for balloon-uncrossable lesions are far from standardized, they may include advanced wire support and anchoring techniques, retrograde access, or default to atherectomy^26^^,^^30^^,^^31^—all of which add time, complexity, and risk. The compatibility of the Javelin IVL catheter with 0.014 standard guidewires mitigates procedural complexity in some cases^33^, ^34^, ^35^, ^36^ and facilitates wire exchange through the over-the-wire port. The generation of energy-based acoustic pressure waves, both in front of and along the side of the catheter tip as it passes through lesions, allows modification of calcified lesions as the catheter crosses. In many cases, the Javelin IVL catheter was used to create a channel to allow for crossing of imaging catheters to better assess lesion characteristics and guide further treatment. The use of IVUS to guide peripheral vascular interventions has been shown in several studies to improve patency and reduce short- and long-term complications.^37^^,^^38^

### Tortuous anatomy and small vessels

In both small vessels and tortuous anatomy, the use of complex calcium-modification tools such as atherectomy is often not recommended owing to higher risk of vascular complications including significant medial and adventitial injury,^16^^,^^17^ as well as the need for embolic protection devices, which may not be deliverable in these scenarios.^19^^,^^39^ Furthermore, small-diameter vessels can limit balloon crossing,^40^^,^^41^ and tortuosity can lead to incomplete balloon rewrap with standard PTA balloons,^13^ which may also preclude the use of balloon-based IVL platforms. Javelin IVL’s low crossing profile (1.5 mm) and lack of a balloon, coupled with a working diameter equivalent to the smallest rotational atherectomy burr,^36^ makes it a potential solution for complex, small caliber, tortuous peripheral vessels.

### Calcific nodules

Nodular calcification remains the most difficult calcium morphology in which to achieve predictably optimal results.^11^^,^^12^ Presence of calcific nodules has been shown to increase the risk for balloon rupture during PTA,^42^^,^^43^ and the same risk may affect balloon-based IVL. Nodular calcification increases in frequency with extent of generalized vascular calcification—especially so in infrapopliteal lesions,^12^^,^^44^ where superficial and medial calcification are highly prevalent.^3^^,^^9^ Javelin IVL’s ability to approximate to the lesion with accuracy and focus energy delivery to these most complex and occlusive lesions could potentially enhance procedural efficiency while avoiding device failure and the risks accompanying atherectomy.^16^, ^17^, ^18^ Combination therapies, such as atherectomy for debulking of nodules followed by IVL for medial calcium modification, have been successful, and there are likely cases where combination calcium modification strategies will be necessary.^45^^,^^46^

### Limitations

Although providing a new option for delivery of IVL, with demonstrated safety and efficacy in complex calcific vascular lesions, the Javelin IVL platform does have contraindications detailed in the instructions for use document as well as potential limitations. The single emitter has a total of 120 pulses, which may sometimes prove insufficient in the treatment of particularly long, diffused lesions, common particularly in the infrapopliteal territory.^7^^,^^8^ Similarly, where reference vessel diameter is large (eg, above-the-knee lesions), combination with balloon-based IVL platforms or other technologies has been used effectively^45^^,^^46^; however, this approach will likely be challenging from an economic standpoint. As noted in the initial pivotal studies, combination with conventional balloon-based IVL platforms was predominantly seen in larger-caliber vessels above the knee and long calcified lesions where Javelin IVL use facilitated crossing of the subsequent calcium-modification devices. In contrast, in BTK lesions, Javelin IVL use alone was mostly sufficient in crossing and modifying many lesions, resulting in final residual stenosis comparable to prior balloon-based IVL studies.^24^^,^^25^

## Conclusions

The Javelin peripheral IVL catheter is designed specifically for the treatment of balloon-uncrossable calcific peripheral arterial lesions, via its single emitter, placed immediately proximal to the tip of the catheter. Scenarios in which Javelin IVL may be particularly applicable include balloon-uncrossable lesions, CTOs, tortuous or small vessel anatomy, and calcific nodules. Continued real-world assessment of the use of this technology will be necessary to understand how it fits into physicians’ preferred algorithms for the treatment of complex peripheral endovascular lesions.

## Author Contributions

Conception and design: AH, SS, MS, NW, JC

Analysis and interpretation: AH, SS, MS, NW, JC

Data collection: AH, SS, MT, AL, JP, JC

Writing the article: AH, SS, NW, JC

Critical revision of the article: AH, SS, MS, MT, AL, JP, NW, JC

Final approval of the article: AH, SS, MS, MT, AL, JP, NW, JC

Statistical analysis: Not applicable

Obtained funding: Not applicable

Overall responsibility: AH

## Funding

The Mini S Feasibility Study (NCT05058456) and FORWARD PAD IDE Study (NCT05858905) with the Shockwave Javelin peripheral intravascular lithotripsy catheter were sponsored by 10.13039/100031956Shockwave Medical Inc. No specific funding was provided for this manuscript.

## Disclosures

A.H. is on the speaker’s bureau and advisory board for Shockwave Medical Inc. M.T., A.L., J.J.P., and N.E.J.W. are employees of Shockwave Medical Inc. J.D.C. is on the advisory board for Shockwave Medical Inc. The remaining authors report no conflicts.
