# Supplementary material for: Device design and applications of the novel JAVELIN peripheral intravascular lithotripsy catheter
Source: J Vasc Surg Cases Innov Tech. 2026 May 15;12(4):102315. doi: 10.1016/j.jvscit.2026.102315 (PMC13285703; doi:10.1016/j.jvscit.2026.102315)
Supplement: Supplementary material [file mmc1.docx]

Supplemental Material 1: Inclusion and Exclusion Criteria

**Inclusion Criteria**

Subjects were required to meet all of the following inclusion criteria in order to be enrolled in the study. For lesion characteristics, each target lesion was required meet eligibility. A maximum of two (2) target lesions could have been treated per subject; target lesions could have been in the same limb or in different limbs.

**General Inclusion Criteria**

1. Age of subject is ≥ 18 years.

2. Subject is able and willing to comply with all assessments in the study.

3. Subject or subject’s legal representative has been informed of the nature of the study, agrees to participate, and has signed the approved consent form.

4. Estimated life expectancy > 1 year.

5. Rutherford Clinical Category 2, 3, 4 or 5 of the target limb(s).

**Angiographic Inclusion Criteria**

6. One or two target lesion(s) located in a native de novo superficial femoral, popliteal or infrapopliteal artery (above the ankle joint), in one or both limbs.

7. Target lesion reference vessel diameter (RVD) between 2.0 mm and 7.0 mm by investigator visual estimate.

8. Target lesion stenosis ≥70% (for vessels below the knee, defined as P3 to the ankle joint) or ≥90% (for vessels above the knee) by investigator visual estimate.

9. Target lesion length is ≤150 mm by investigator visual estimate. Target lesion can be all or part of the 150 mm treated zone.

10. Calcification is at least moderate defined as presence of fluoroscopic evidence of calcification: 1) on parallel sides of the vessel and 2) extending > 50% the length of the lesion if lesion is ≥50mm in length; or extending for minimum of 20mm if lesion is <50mm in length.

**Exclusion Criteria**

Subjects who meet any of the following exclusion criteria may not be enrolled in the study:

**General Exclusion Criteria**

1. Rutherford Clinical Category 0, 1 and 6 (target limb).

2. History of endovascular or surgical procedure on the target limb within the last 30 days, or planned within 30 days of the index procedure, with the exception of toe amputation. Note: inflow treatment of non-target lesions is allowed providing successful treatment.

3. Subject in whom antiplatelet or anticoagulant therapy is contraindicated.

4. Subject has known allergy to contrast agents or medications used to perform endovascular intervention that cannot be adequately pre-treated.

5. Subject has known allergy to urethane, nylon, or silicone.

6. Myocardial infarction within 60 days prior to enrollment.

7. History of stroke within 60 days prior to enrollment.

8. Subject has acute or chronic renal disease with eGFR <30 ml/min/1.73 m^2^ (using CKD-EPI formula), unless on renal replacement therapy.

9. Subject is pregnant or nursing.

10. Subject is participating in another research study involving an investigational agent (pharmaceutical, biologic, or medical device) that has not reached the primary endpoint.

11. Subject has other medical, social or psychological problems that, in the opinion of the investigator, preclude them from receiving this treatment, and the procedures and evaluations pre- and post-treatment.

12. Covid-19 diagnosis within 30 days.

13. Planned use of cutting/scoring balloons, re-entry or atherectomy devices in target lesion(s) during the index procedure.

14. Planned major amputation of target limb.

15. Acute limb ischemia.

16. Occlusion of all the inframalleolar outflow arteries/vessels (i.e., desert foot).

17. Subject already enrolled into this study.

**Angiographic Exclusion Criteria**

18. Failure to successfully treat clinically significant inflow lesions in the ipsilateral iliac, femoral, or popliteal arteries, defined as ≤30% residual stenosis with no serious angiographic complications (e.g. embolism).

19. Failure to successfully treat significant non-target infrapopliteal lesions, if treated prior to treatment of target lesion(s). Successful treatment is defined as obtaining ≤50% residual stenosis with no serious angiographic complications (e.g., embolism).

20. Target lesion includes in-stent restenosis.

21. Evidence of aneurysm or thrombus in target vessel.

22. No calcium or mild calcium in the target lesion.

23. Target lesion within native or synthetic vessel grafts.

24. Failure to successfully cross the guidewire across the target lesion; successful crossing defined as tip of the guidewire distal to the target lesion in the absence of flow limiting dissections or perforations.
